# Conservation of Resources Theory: Technology and Caregiver Strain

**DOI:** 10.1093/geroni/igaa057.1515

**Published:** 2020-12-16

**Authors:** Shinduk Lee, Marcia Ory, Deborah Vollmer Dahlke, Tiffany Shubert, Steve Popovich, Matthew Smith

**Affiliations:** 1 Texas A&M University, College Station, Texas, United States; 2 DVD Associates, LLC, Austin, Texas, United States; 3 Shubert Consulting, Chapel Hill, North Carolina, United States; 4 Clairvoyant Network, Austin, Texas, United States

## Abstract

Using the Conservation of Resources Theory, this study examined how caregiver strain was influenced by care recipients’ use of falls alert wearables. Online survey data from 486 unpaid caregivers for adults aged 50 and older were analyzed. Structural equation modeling was used to test the following hypotheses: (1) caregivers with fewer financial resources would engage in fewer resource conservation strategies (e.g., care recipients’ use of falls alert wearables); (2) resource conservation strategy engagement would be associated less resource loss; and (3) the effect of resource conservation strategies on caregiver strain would be less salient than the effect of resources used on caregiving (e.g., time and social support). The hypothesized model had a good model fit (CFI=.910), with SRMR (.060) and RMSEA (.062) being close to .05. All hypothesized paths were statistically-significant, except for the direct effect of using falls alert wearables on social support (p=.076) and caregiver strain (p=.135). As hypothesized, higher income was associated with greater likelihood of using falls alert wearables (b=.10, p>.022). Technology use was associated with less time spent on caregiving (b=-.16, p<.001) and had statistically-significant indirect effects on caregiver strain (b=-.03, p=.008). The total effect of using falls alert wearables (b=.04, p=.394) on caregiver strain was less powerful than the effect of time (b=.20, p<.001) or social support (b=-.28, p<.001). Study findings suggest the benefits of using falls alert wearables to alleviate time-related burdens and downstream caregiver strain among unpaid caregivers. Future efforts should investigate the relative advantage of wearables for other caregiving purposes.

